# MiR-223-3p functions as a tumor suppressor in lung squamous cell carcinoma by miR-223-3p-mutant p53 regulatory feedback loop

**DOI:** 10.1186/s13046-019-1079-1

**Published:** 2019-02-12

**Authors:** Peng Luo, Qi Wang, Yuanyuan Ye, Ju Zhang, Dapeng Lu, Longqiang Cheng, Hangcheng Zhou, Mingran Xie, Baolong Wang

**Affiliations:** 1 Department of Clinical Laboratory, The First Affiliated Hospital of University of Science and Technology of China, Hefei, China; 2 Anhui Medical University, Hefei, China; 3 School of Life Sciences, University of Science and Technology of China, Hefei, China; 4 Department of Pathology, The First Affiliated Hospital of University of Science and Technology of China, Hefei, China; 5 Department of Thoracic Surgery, The First Affiliated Hospital of University of Science and Technology of China, Hefei, China; 6 Department of Clinical Laboratory, Division of Life Sciences and Medicine, The First Affiliated Hospital of USTC, University of Science and Technology of China, Hefei, Anhui 230001 People’s Republic of China

**Keywords:** Mutant p53-miR-223-3p-feedback loop-lung squamous cell carcinoma

## Abstract

**Background:**

MicroRNAs have an important role in diverse biological processes including tumorigenesis. MiR-223 has been reported to be deregulated in several human cancer types. However, its biological role has not been functionally characterized in lung squamous cell carcinoma (LSCC). The following study investigates the role of miR-223-3p in LSCC growth and metastasis and its underlying mechanism.

**Methods:**

MicroRNA profiling analyses were conducted to determine differential miRNAs expression levels in LSCC tumor tissues that successfully formed xenografts in immunocompromised mice (XG) and failed tumor tissues (no-XG). RT-PCR and in situ hybridization (ISH) was performed to evaluate the expression of miR-223-3p in 12 paired adjacent normal tissues and LSCC specimens. Cell proliferation and migration were assessed by CCK-8, colony formation and Transwell assay, respectively. The role of miR-223-3p in LSCC tumorigenesis was examined using xenograft nude models. Bioinformatics analysis, Dual-luciferase reporter assays, Chromatin immunoprecipitation (ChIP) assay and Western blot analysis were used to identify the direct target of miR-223-3p and its interactions.

**Results:**

MiR-223-3p was downregulated in LSCC tissues that successfully formed xenografts (XG) compared with tumor tissues that failed (no-XG), which was also significantly reduced in LSCC tissues compared with the adjacent normal tissues. Gain- and loss-of function experiments showed that miR-223-3p inhibited proliferation and migration in vitro. More importantly, miR-223-3p overexpression greatly suppressed tumor growth in vivo. Mechanistically, we found that mutant p53 bound to the promoter region of miR-223 and reduced its transcription. Meanwhile, p53 is a direct target of miR-223-3p. Thus, miR-223-3p regulated mutant p53 expression in a feedback loop that inhibited cell proliferation and migration.

**Conclusions:**

Our study identified miR-223-3p, as a tumor suppressor gene, markedly inhibited cell proliferation and migration via miR-223-3p-mutant p53 feedback loop, which suggested miR-223-3p might be a new therapeutic target in LSCC bearing p53 mutations.

**Electronic supplementary material:**

The online version of this article (10.1186/s13046-019-1079-1) contains supplementary material, which is available to authorized users.

## Background

Lung cancer is a leading cause of cancer-related deaths worldwide. Non-small cell lung cancer (NSCLC) accounts for 85% of all lung cancer cases. In addition, more than 65% of patients with NSCLC are diagnosed with an advanced or metastatic disease and have a 5-year survival rate that is less than 20% [1, 2]. Lung squamous cell carcinoma (LSCC) is the second most common type of NSCLC, accounting for more than 30% of NSCLC [3]. So far, no molecularly targeted agents have been specifically developed for its treatment [4, 5]. Therefore, a detailed study of the development and progression of LSCC is essential for improving the diagnosis, prevention, and treatment of this disease.

MicroRNAs (miRNAs) are small (18–25 nucleotides) noncoding RNAs that negatively regulate gene expression mainly through the 3′-untranslated region (3’UTR) [6]. They have been implicated in a variety of biological processes, such as cell proliferation, invasion, and drug sensitivity of tumors [7, 8]. Lung carcinogenesis is a multistep process. Although microRNA profiling has contributed to the understanding of the biology of NSCLC, the microRNA signatures identified in NSCLC (including adenocarcinoma and squamous cell carcinoma) were not consistent among different studies [9]. Moreover, miRNAs have different effects on different tumor subtypes [10, 11]. Therefore, still many questions remain regarding the exact mechanisms, and biological functions of miRNAs in the LSCC subtypes.

Patient-derived tumor xenografts (PDTX), which are xenograft models developed by transplanting human tumors directly into immunocompromised mice, have been suggested as a very realistic preclinical cancer model [12–15]. Tumors that successfully form xenografts are biologically more aggressive and may be more representative of cancers with a higher propensity to relapse after surgery [16, 17]. Thus, in order to identify the miRNAs with greatest potential to affect tumor proliferation and metastasis, the present study investigated the miRNAs of LSCC tissues that successfully formed a xenografts (XG) compared with tumor tissues that failed to form xenografts (no-XG).

Here we reported that miR-223-3p, which was down regulated in the XG group, was also significantly reduced in LSCC tissues compared with the adjacent normal tissues. MiR-223-3p potently suppressed tumor proliferation and migration in vitro. Importantly, miR-223-3p overexpression greatly suppressed tumor growth in vivo. Our results further demonstrated that mutant p53 bound to the promoter region of miR-223 and reduced its transcription in LSCC bearing p53 mutations. Meanwhile, we provide evidence that miR-223-3p directly targets mutant p53, to suppress cell proliferation and migration. Collectively, the results of this study provide an explanation for the aggressiveness of LSCC and this is a mutual regulation between miR-223-3p and mutant p53. Our results also suggest that miR-223-3p might be a new therapeutic target in LSCC bearing p53 mutations.

## Methods

### Tissue specimens and cell culture

Twelve primary human LSCC tissues and normal lung specimens were obtained from patients who underwent surgery at the First Affiliated Hospital of University of Science and Technology of China (Anhui Provincial Hospital). Each cancer specimen contained at least 80% tumor cells, as confirmed by the microscopic examination. Tissues were preserved by snap-freezing approach and were stored at − 80 °C for subsequent protein and RNA test. Tumor samples and clinical records were obtained from patients after they have signed informed consent. Moreover, the Anhui Provincial Hospital Ethical Committee approved this study.

Human NSCLC cell lines (SK-MES-1, NCI-H520 and NCI-H2170) were obtained from the Chinese Academy of Sciences Cell Bank (Shanghai, China). SK-MES-1 cells were maintained in Dulbecco’s modified Eagle’s medium (DMEM), while the rest of the cell lines were grown in RPMI 1640 medium containing 10% fetal bovine serum and were cultured at 37 °C in 5% CO_2_.

### Establishment of PDTX models

The fresh tumor samples were placed into sterile Petri dishes, washed three times with phosphate-buffered saline (PBS), and cut into 3 mm^3^ fragments. Tumor fragments were implanted subcutaneously into the left and right flanks of mice (3–5 mice/patient specimen) and were weekly monitored using calipers when the implanted tissue was palpable. The tumor volume was calculated using following formula: (length×width^2^)/2. When the volume of tumor reached 500 mm^3^, LSCC tissues was considered successful transplantation.

### MicroRNA microarray

After RNA isolation from the samples, the miRCURY Hy3/Hy5 Power Labeling Kit (Exiqon, Vedbaek, Denmark) was used for miRNA labeling according to the manufacturer’s instructions. Expressed data were normalized using the median normalization. After normalization, significant differentially expressed miRNAs were identified through Volcano plot filtering. Hierarchical clustering was performed using MEV software (v4.6, TIGR). Differentially expressed miRNAs with statistical significance between the two groups were identified through *P* value/FDR filtering (*P* < 0.05).

### Transfection of cell lines

Transient and stable transfection experiments in cells were used. LV-mutant p53^215C > G^ and corresponding control lentivirus (LV-NC) were designed and purchased from GenePharma (Shanghai, China). LV-mutant p53^215C > G^ and LV-NC were initially proved by DNA sequencing before transfection. Lentivirus was used to infect SK-MES-1 cells with an appropriate multiplicity of infection (MOI). The stable overexpression cell lines were generated by selecting transfected cells in complete culture medium containing puromycin for at least 14 days. The cell transient transfection was performed using Lipofectamine 2000 (Invitrogen, CA, USA), according to the manufacturer’s protocol. The media was changed 4–6 h after transfection without washing with phosphate-buffered saline (PBS). The miR-223-3p mimics (sense 5’-UGUCAGUUUGUCAAAUACCCCA-3′ and antisense 5’-GGGUAUUUGACAAACUGACAUU-3′), mimics-NC (sense 5’-UUCUCCGAACGUGUCACGUTT-3′ and antisense 5’-ACGUGACACGUUCGGAGAATT-3′), miR-223-3p inhibitors (5’-CAGUACUUUUGUGUAGUACAA-3′), inhibitors-NC (5’-CAGUACUUUUGUGUAGUACAA-3′), siRNAs (siRNA-p53–1; sense 5’-GCUGUGGGUUGAUUCCACATT-3′ and antisense 5’-UGUGGAAUCAACCCACAGCTT-3′, and siRNA-p53–2; sense 5’-CCACCAUCCACUACAACUATT-3′ and antisense 5’-UAGUUGUAGUGGAUGGUGGTT-3′, and NC (sense 5’-UUCUCCGAACGUGUCACGUTT-3′ and antisense 5’-ACGUGACACGUUCGGAGAATT-3′) .

### Quantitative RT-PCR

Total RNA was extracted using TRIzol reagent (Invitrogen) according to the manufacturer’s protocol. For analyzing miRNA expression, miRNA was reverse-transcribed using the Mir-XTM miRNA First-Strand Synthesis Kit (TaKaRa, Dalian, China) under the following conditions: 37 °C for 60 min, 85 °C for 5 min, and were held at 4 °C. qPCR reactions were performed using the SYBR Premix Ex Taq II kit (TaKaRa) and detected on the ABI 7500 Real-Time PCR system. qPCR was performed under the following conditions: 95 °C for 5 min, 95 °C for 5 s, and 60 °C for 34 s for 40 cycles. U48 were used as loading controls for the quantitation miRNAs. The primers of miR-223-3p and U48 were purchased from GeneCopoeia (Guangzhou, China). The levels of gene expression were calculated using the 2^-ΔΔCT^ methods. The data were representative of three independent experiments that were performed in different days.

### Cell survival assays

The effects of miRNA-223-3p expression on the proliferation of LSCC cells were assessed using the Cell Counting Kit-8 (CCK-8; Kumamoto, Japan). Briefly, the cells were transfected for 24 h and plated on 96-well plates. Subsequently, CCK-8 was added to each well at various times and incubated at 37 °C for 1.5 h. The absorbance at 450/630 nM was measured using a microplate spectrophotometer (Tecan Group Ltd., Männedorf, Switzerland). A minimum of five wells were assessed for each group.

### Colony formation assay

For colony formation assays, 1 × 10^3^ cells were inoculated into 6-well plates with 2 ml of medium containing 10% fetal bovine serum (FBS). After 14 days, the resulting colonies were rinsed with PBS, fixed with 4% formaldehyde for 10 min, stained with 0.1% crystal violet for 30 min, and then rinsed with PBS again. Finally, the number of clones was counted to evaluate cell proliferation.

### Cell apoptosis analysis

Cells were transfected with control or miR-223-3p mimic. Apoptosis was assessed by measuring the membrane redistribution of phosphatidylserine using an Annexin V–PI apoptosis detection kit (Becton Dickinson).

### In vitro migration assays

Cell migration was detected using Transwell chambers (8 mm, Corning Costar Co., USA). After the treatment, the cells were seeded in serum-free media on the upper side of a Transwell chamber. The cells were allowed to migrate toward the media containing 10% FBS for 24 h. After the incubation period, the cells on the lower side of the membrane were fixed, stained with crystal violet, and counted. The migration indices were calculated as the mean number of cells in 10 random fields at × 100 magnification.

### In vivo tumor model

Balb/c female nude mice, 6–8 week old, weighing 20–25 g, were obtained from the Shanghai SLAC Laboratory Animal Center (Shanghai, China). All the animals were housed in an environment with temperature of 22 ± 1 °C, relative humidity of 50 ± 1% and a light/dark cycle of 12/12 h. All animal studies (including the mice euthanasia procedure) were done in accordance with institutional guidelines and in compliance with national and international laws and policies.

Mice were subcutaneously injected with LSCC patients-derived tumor tissues. When tumor volume reached 50–100 mm^3^, the mice were randomly assigned to treatment or control group. MiR-223-3p agomir (5 nmol) or agomir-NC was given locally by direct injection into the xenografts twice a week for 3 weeks. The tumors were monitored every 3 days, and tumor volumes were calculated using the following formula: 1/2 × length × (width)^2^. After 21 days, the mice were euthanized, necropsies were performed, and tumors were weighted. The primary tumors were excised, and tumor tissues were used to perform IHC staining, ISH staining and qRT-PCR.

### Western blot

Equal amounts of proteins collected from different types of cell lysates were fractionated using sodium dodecyl sulfate–polyacrylamide gel electrophoresis and were then transferred onto a polyvinylidene fluoride membrane (pore size; 0.45 mm). The membrane was blocked for 2 h with 5% non-fat milk at room temperature and was then incubated with primary antibodies: p53 (1:1000 dilution, Proteintech), β-actin (1:1000 dilution, Proteintech), or GAPDH (1:1000 dilution, Proteintech) at 4 °C overnight. Membranes were then washed and incubated with secondary antibodies at room temperature for 2 h. The blot was processed using an enhanced chemiluminescence kit (Santa Cruz). All experiments were run in triplicate.

### Immunohistochemical (IHC) assay

Paraffin sections were reacted with p53 (1:100 dilution), Ki-67(1:100 dilution) overnight at 4 °C, and then incubated with the corresponding secondary antibodies. The reactions were developed using the DAB Kit (BD Bioscience, San Jose, CA, USA), and the sections were counterstained with hematoxylin.

### In situ hybridization (ISH) analysis

Human LSCC tissues and normal lung specimens were fixed in 4% paraformaldehyde and then were embedded in paraffin. A FAM-labeled miR-223-3p oligonucleotide probes (5′-TGTCAGTTTGTCAAATACCCCA-3′) were obtained from Sangon Biotech (Shanghai, China). Slides were deparaffinized and incubated for 25 min at room temperature with Protease K; subsequently, the sections were prehybridized in a humid chamber at 37 °C for 1 h. Then, the tissues were hybridized with a miR-223-3p probe at 37 °C overnight. After hybridization, the slides were washed with graded-diluted sodium citrate buffer (SSC) at 37 °C for 30 min; subsequently, the sections were stained with DAPI for 8 min. Finally, the tissues were imaged.

### p53 mutation analysis

Since p53 mutations occur throughout the gene in human cancer, the p53 coding sequence was screened for mutations using Sanger sequencing. The GeneBank accession number U94788.1 was used. The three primers for p53 were as follows: (1) forward 5’CTTTCCACGACGGTGACACG3’ and reverse 5’-CTTCCACTCGGATAAGATGCTGA-3′; (2) forward 5’-CCATCTACAAGCAGTCACAGCAC-3′ and reverse 5’-CAAATGGAAGTCCTGGGTGC-3′; (3) forward 5’-CATCTACAAGCAGTCACAGCAC-3′ and reverse 5’-CCAAACATCCCTCACAGTAAAA-3′. Amplified products were confirmed by electrophoresis on 1.5% agarose gels. PCR products were purified using the SanPrep Column DNA Gel Extraction Kit (Sangon Biotech, Shanghai, China) and were sequenced on an ABI PRISM 3730XL Genetic Analyzer (Applied Biosystems, CA, USA).

### Chip assay

SK-MES-1 and NCI-H520 cells were cross-linked with 1% formaldehyde for 15 min at room temperature. The chromatin fragments ranging between 200 and 1000 bp were obtained by sonication. The protein-DNA complex was precipitated by anti-p53 antibody or anti-IgG antibody at 4 °C overnight, and the antibody-protein-DNA complex was collected by protein beads. After being eluted from the beads, the antibody-protein-DNA complex was reversely cross-linked by incubation at 65 °C with 200 mM NaCl. The amount of immunoprecipitated DNA was detected by semiquantitative-PCR. The sequences of primer used in PCR experiments were as follows: sense 5′- GCATCCAGATTTCCGTTGGCTAAC-3′, antisense 5’-GCAAATGGATACCATACCTGTCAGTG -3′.

### Luciferase reporter assay

HEK-293 T cells grown in a 96-well plate, wild-type luciferase reporter (WT-psiCHECK-2), and mutant luciferase reporter (MUT-psiCHECK-2) were co-transfected with miR-223-3p mimics or its corresponding negative control. Firefly and Renilla luciferase activity were measured in cell lysates using a Dual-Luciferase Reporter Assay System (Promega), according to the manufacturer’s protocol.

### Statistical analysis

Numerical data were presented as mean ± standard deviation (SD). All statistical analyses were performed using SPSS 16.0 software. Statistical differences between two groups were determined using the Student’s *t* test. *P* value < 0.05 was considered statistically significant.

## Results

### Identification of miRNAs with the potential to affect tumor proliferation and metastasis

**Fig. 1:**
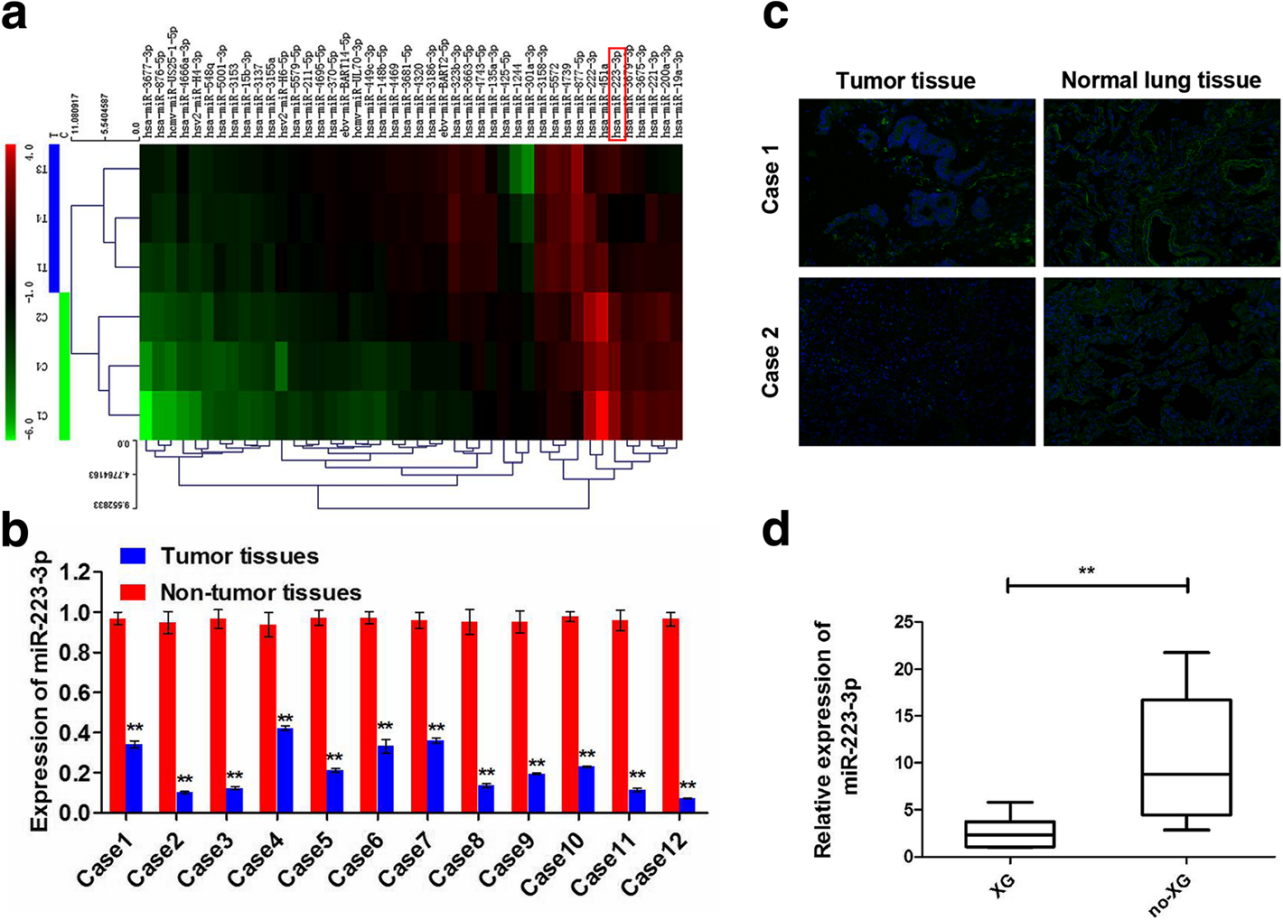
Downregulation of miR-223-3p promoted lung squamous cell cancer development. **a** Microarray analysis of miRNAs from different LSCC tissues that successfully formed xenografts (XG, *n* = 3) and tumor tissues that failed to form xenografts (no-XG, *n* = 3) were presented in a heatmap. Each column represents a microRNA, and each row represents a sample. The relative expression values are depicted according to the color scale. Red indicates high relative expression, and green indicates low relative expression. T: XG tumor tissues; C: no-XG tumor tissues. **b** qRT-PCR analysis of miR-223-3p expression of LSCC tissues and adjacent normal tissues. **c** Representative images of the ISH staining analyses of two different LSCC tissues and adjacent normal tissues using anti-miR-223-3p probe. **d** Compared with no-XG group, the miR-223-3p expression in XG group was significantly decreased. Each qRT-PCR experiment was performed in triplicate. **P < 0.01

Patient-derived tumors that could be engrafted to immunocompromised mice were biologically more aggressive, proliferative, and with a higher propensity to relapse after surgery. To identify the miRNAs with greatest potential to affect tumor proliferation and metastasis, we analyzed miRNAs from three LSCC samples that successfully formed xenografts in immunocompromised mice (XG) and compared them with miRNAs from the other three LSCC samples that failed to form xenografts (no-XG). Using miRNA array, we identified 38 miRNAs that were significantly dysregulated in XG when compared with their expression in no-XG (Fold change> 2, *p* < 0.05). Among them, 27 were up-regulated, and 11 were down-regulated (Fig. 1a). Among these miRNAs, miR-451a [18, 19], miR-222 [20], miR-223 [10, 11, 21], and miR-200a-3p [22] were highly involved in the regulation of tumor growth and migration. Paradoxically, miR-223 was revealed to function as a tumor suppressor in Lewis lung carcinoma cells [10] while miR-223 may function as an oncogene in lung adenocarcinoma A549 cells [11]. Thus, we focused on miR-223-3p for further study in LSCC. Compared with the no-XG group, miR-223-3p was dramatically down-regulated in the XG group, and the expression of miR-223-3p was further measured by qRT-PCR in 12 LSCC tissues (6 successfully formed xenografts and remaining 6 failed to form xenografts) and their corresponding adjacent normal lung tissues. RT-PCR results showed that the miR-223-3p expression was down-regulated in the LSCC tissues compared with that in the normal lung tissues (Fig. 1b). In situ hybridization (ISH) staining further confirmed significantly lower miR-223-3p expression in LSCC tissues compared to that of the adjacent normal lung tissues (Fig. 1c). Moreover, compared with no-XG, miR-223-3p expression in XG was significantly decreased (Fig. 1d).

Clinical materials of 12 LSCC patients and histopathology of their tumors were shown in Table 1. Among them, 5 of 6 patients harboring p53 mutations were successfully formed xenografts. However, 5 of 6 patients harboring wild-type p53 were failed to form xenografts (no-XG). Furthermore, 4 of 5 patients harboring p53^215C > G^ missence mutation were successfully formed xenografts. The results suggested xenograft engraftment was associated with p53 mutations, especially p53^215C > G^ mutation. Altogether, these results revealed the potential possibilities of miR-223-3p and p53 mutations in tumor formation.

**Table 1:** Clinical material of 12 lung squamous cell carcinoma patients and histopathology of their tumors

Cases	Age/sex	TNM stage	Differentiation	TP53 mutant	Xenograft formation forformation
1	69/M	T2N0M0	Moderate	No	No
2	73/M	T2N2M0	Moderate	215C > G, 614A > G	Yes
3	70/M	T4N0M0	Poor	215C > G, 473G > T	Yes
4	75/M	T2N0M0	Moderate	485 T > G	No
5	72/M	T2N0M0	Poor	No	No
6	59/M	T2N0M0	Moderate	No	No
7	74/M	T1N0M0	Poor	No	No
8	58/M	T4N2M0	Poor	215C > G	Yes
9	65/M	T3N0M0	Poor	215C > G, 701A > G	Yes
10	71/M	T2N0M0	Moderate	No	No
11	73/M	T3N0M0	Moderate	No	Yes
12	61/M	T3N2M0	Poor	485 T > G	Yes

### MiR-223-3p repressed the proliferation and migration of LSCC in vitro

**Fig. 2:**
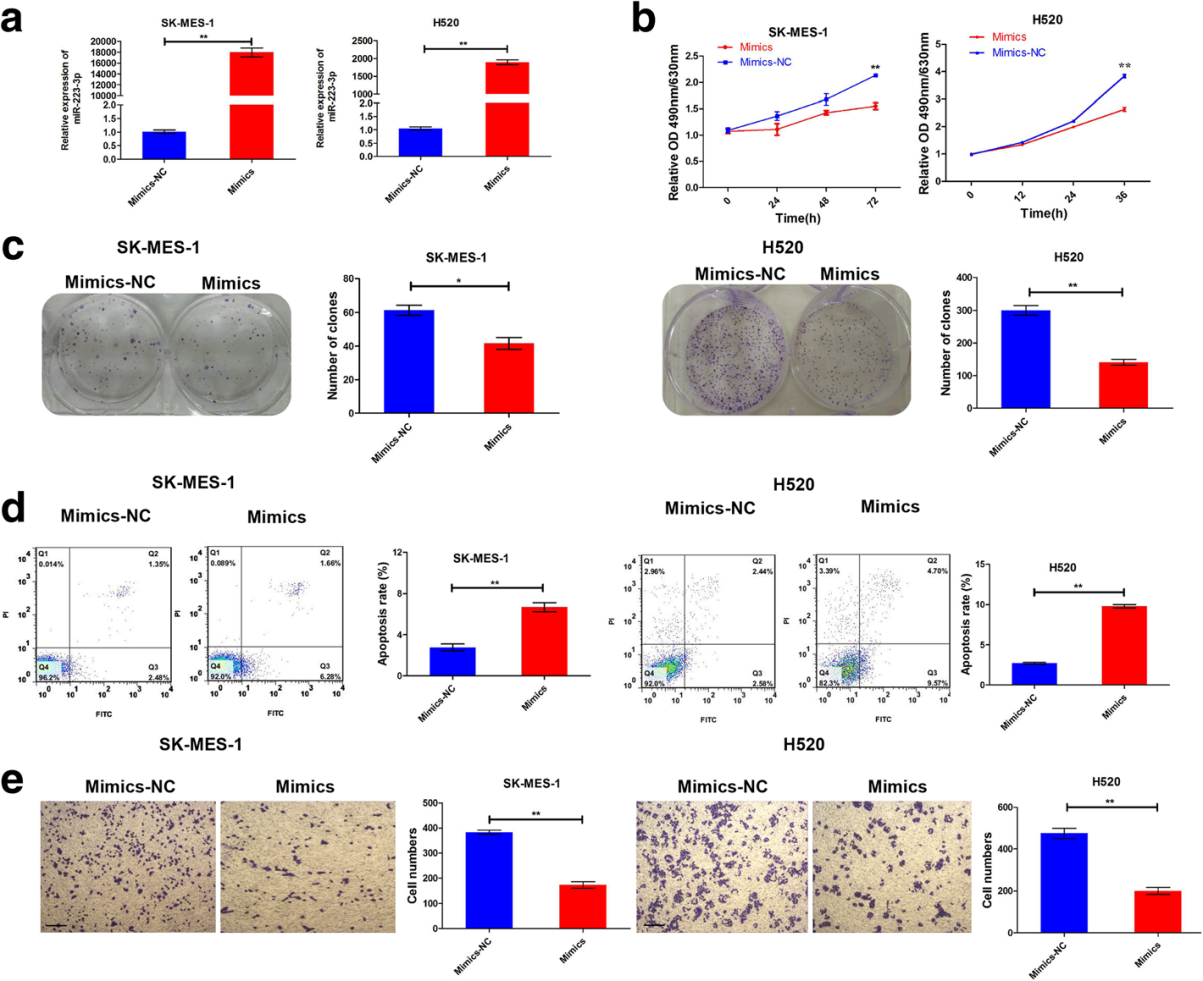
MiR-223-3p suppressed cell proliferation and migration in vitro. **a** Effect of miR-223-3p-mimic transfection into LSCC cells was confirmed using qRT-PCR. Tumor cells were transfected with miR-223-3p mimics or mimics-NC and then subjected to cell viability assay **b**, colony-formation assay **c**, apoptosis analysis **d** and migration assays **e**. The scale bar was 200 μm. Data are presented as the mean ± SD of three replicates. **P < 0.01; *P < 0.05

To identify the function of miR-223-3p in LSCC, SK-MES-1 and NCI-H520, cell lines were transfected with miR-223-3p mimics or negative control (NC), and miR-223-3p levels of transfected cell lines were tested by qRT-PCR (Fig. 2a). Furthermore, CCK-8 assays were performed to measure the effect of miR-223-3p on cell proliferation; significantly suppressed cell viability was observed in SK-MES-1 and NCI-H520 cells transfected with miR-223-3p mimics compared with NC (Fig. 2b). Similarly, miR-223-3p mimics were identified to exhibit a significantly inhibited colony-forming ability, as demonstrated by the decrease in colony numbers (Fig. 2c). Correspondingly, miR-223-3p knockdown by the inhibitor led to a significant increase in cell viability and colony numbers in SK-MES-1 and NCI-H520 cells (Additional file 1: Figure S1a-c). In addition, flow cytometry (FCM) indicated that miR-223-3p mimics significantly promoted cell apoptosis (Fig. 2d). To further study whether the migration ability of LSCC cells was affected by miR-223-3p, Transwell assays were performed. The results indicated that miR-223-3p mimics could significantly inhibit the migration of SK-MES-1 and NCI-H520 cells (Fig. 2e) whereas miR-223-3p inhibition promoted the migration (Additional file 1: Figure S1d). Taken together, these results suggest that miR-223-3p harbors the ability to suppress the proliferation and migration of LSCC cells in vitro.

### Mutant p53 negatively regulated miR-223-3p expression

**Fig. 3:**
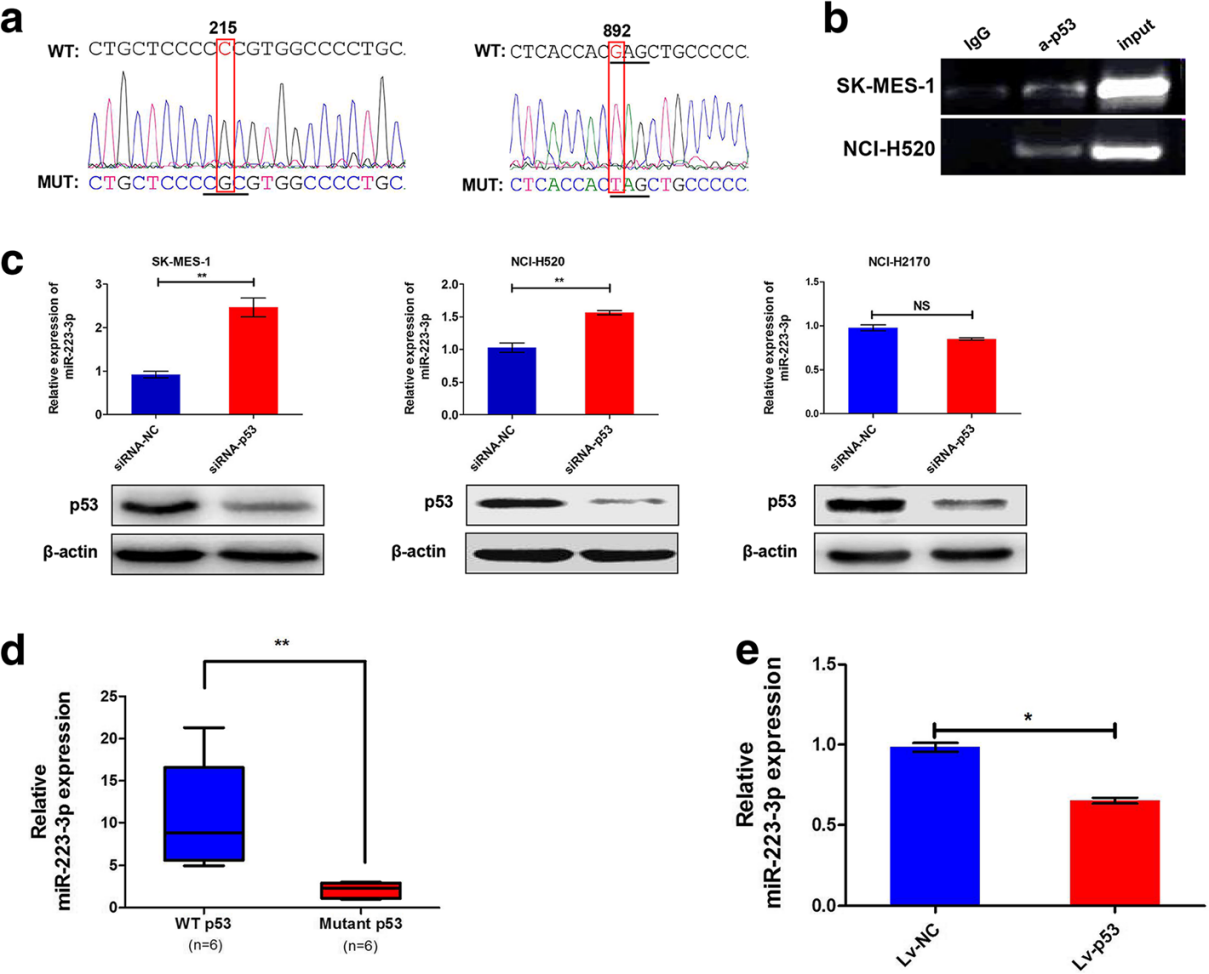
Mutant p53 negatively regulated miR-223-3p expression. **a** The p53 sequencing analysis found LSCC cells (NCI-H520, SK-MES-1) bearing missense (215C > G) and nonsense (892G > T) mutations. **b** ChIP analysis revealed direct binding of mutant p53 to the miR-223 promoter. **c** Downregulation of p53 in SK-MES-1 and NCI-H520 cells increased miR-223-3p expression but not in NCI-H2170 with wild type p53. **d** qRT-PCR results showing that miR-223-3p was significantly down-regulated in the LSCC tissues with mutant p53 compared with the LSCC tissues with wild type p53. **e** Mutant p53^215C > G^ overexpression significantly decreased miR-223-3p expression. **P < 0.01; *P < 0.05; NS: No statistical significance

Next, further studies were made to investigate the underlying mechanism for miR-223-3p downregulation in LSCC. In fact, Masciarelli et al. reported that mutant p53 proteins down-regulated miR-223 expression in breast and colon cancer cell lines by binding miR-223 promoter and reducing its transcriptional activity [23]. Experiments were carried out to explore whether downregulation of miR-223-3p expression in LSCC could be modulated by mutant p53. The p53 gene was sequenced and analyzed. The p53 sequence analysis found that SK-MES-1 cells had missense (215C > G) and nonsense (892G > T) mutations, and that NCI-H520 cells had missense (215C > G) mutation (Fig. 3a). ChIP assay revealed that mutant p53 directly binds to the miR-223 promoter in SK-MES-1 and NCI-H520 cells (Fig. 3b). We thus silenced mutant p53 in the SK-MES-1 and NCI-H520 by siRNA, and then evaluated miR-223-3p expression. MiR-223-3p was up-regulated upon transient silence of mutant p53. Yet, downregulation of p53 expression had no effect on miR-223-3p expression in NCI-H2170 cells (wild type p53) (Fig. 3c). In addition, compared to the LSCC tissues with wild-type p53, the relative expression level of miR-223-3p did significantly decrease in the LSCC tissue samples with mutant p53 (Fig. 3d). Moreover, miR-223-3p was significantly down-regulated upon mutant p53^215C > G^ upregulation in the SK-MES-1 (Fig. 3e). These observations indicated that miR-223-3p was regulated by mutant p53 at the transcriptional level.

### MiR-223-3p directly targeted p53

**Fig. 4:**
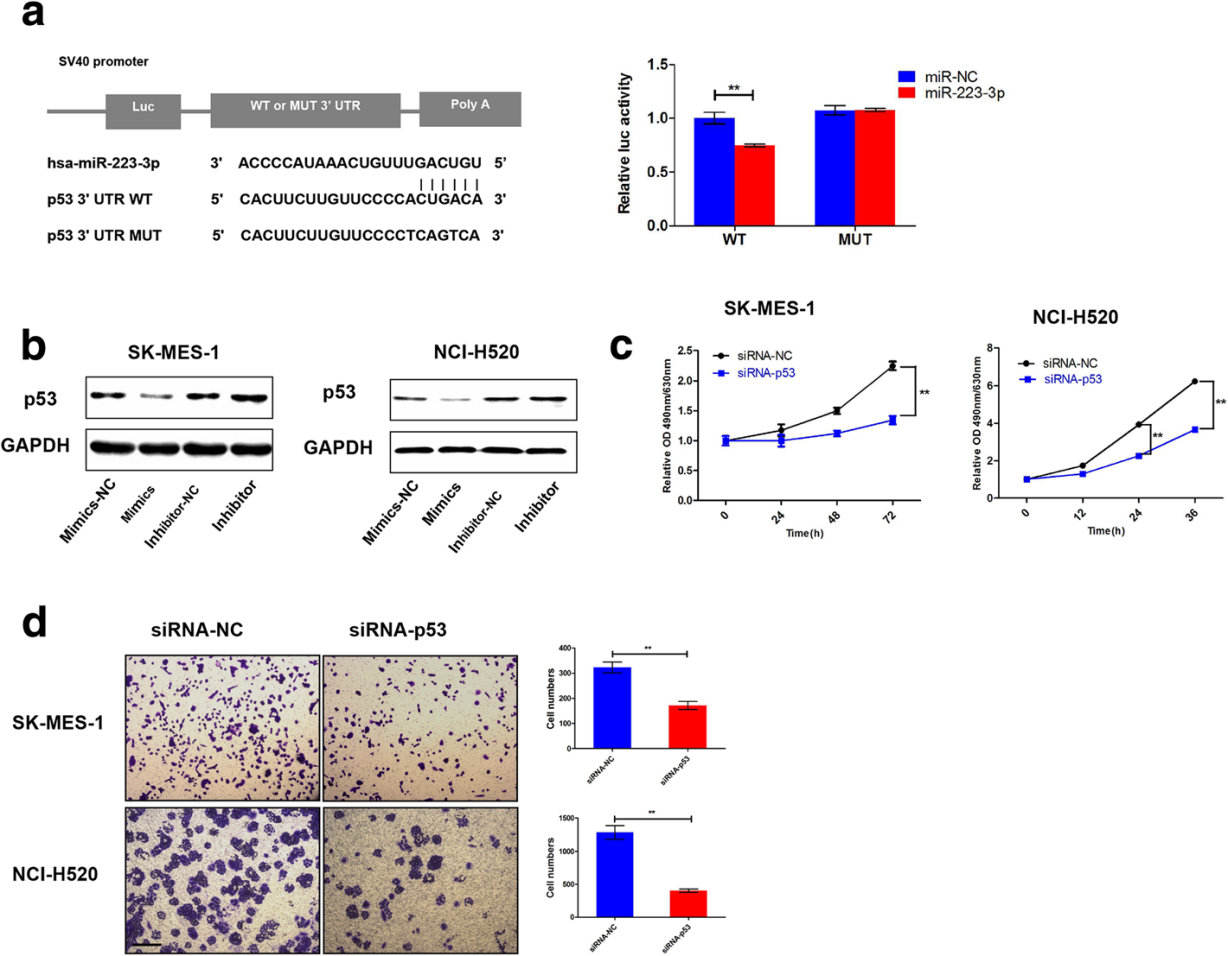
p53 was a target of miR-223-3p. **a** The putative miR-223-3p binding site in the p53 3’-UTR. The luciferase activity was analyzed after co-transfection with either miR-223-3-mimics or the negative control with the psiCHECK-p53 wild-type plasmid or mutant plasmid in 293 T cells. **b** p53 protein levels were determined using Western blot analysis after transfection of miR-223-3p mimic, mimic-NC, inhibitor or inhibitor-NC into LSCC cells. **c** p53 downregulation significantly suppressed the in vitro growth of the LSCC cells in a CCK-8 assay. **d** The transwell assay showed that p53 knockdown markedly decreased the migratory potential of the LSCC cells. These results are representative of at least three independent experiments. All bars represent the mean values ± SD. The scale bar was 200 μm. **P < 0.01

To elucidate how miR-223-3p inhibits cell proliferation and migration in LSCC harboring p53 mutant. MiRNA target-predicted database (http://www.targetscan.org) showed that p53 is a direct target of miR-223-3p. Then, we performed a luciferase reporter assay to confirm that miR-223-3p directly binds to the 3′ untranslated region (UTR) of p53. Our results showed that overexpression of miR-223-3p significantly reduced luciferase activity of the reporter gene in wild type, but not in mutant type, indicating that miR-223-3p directly targeted the p53 3’-UTR (Fig.4a). Consistent with the results of the reporter assay, transfection with miR-223-3p mimics resulted in a significant decrease in p53 protein level in SK-MES-1 and NCI-H520 by western blot. Furthermore, p53 expression was significantly increased by transfection with miR-223-3p inhibitor (Fig. 4b**)**. In addition, similar to miR-223-3p mimics, the downregulation of p53 significantly inhibited the proliferation and migration, which was measured by CCK-8 and Transwell assays (Fig. 4c and d). These results indicate that miR-223-3p inhibits cell proliferation and migration by targeting and suppressing mutant p53 in LSCC. All together, the aforementioned observations indicated that miR-223-3p and p53 were reciprocally linked in a feedback loop in LSCC bearing p53 mutations.

### MiR-223-3p suppressed the proliferation of LSCC in the nude mice

**Fig. 5:**
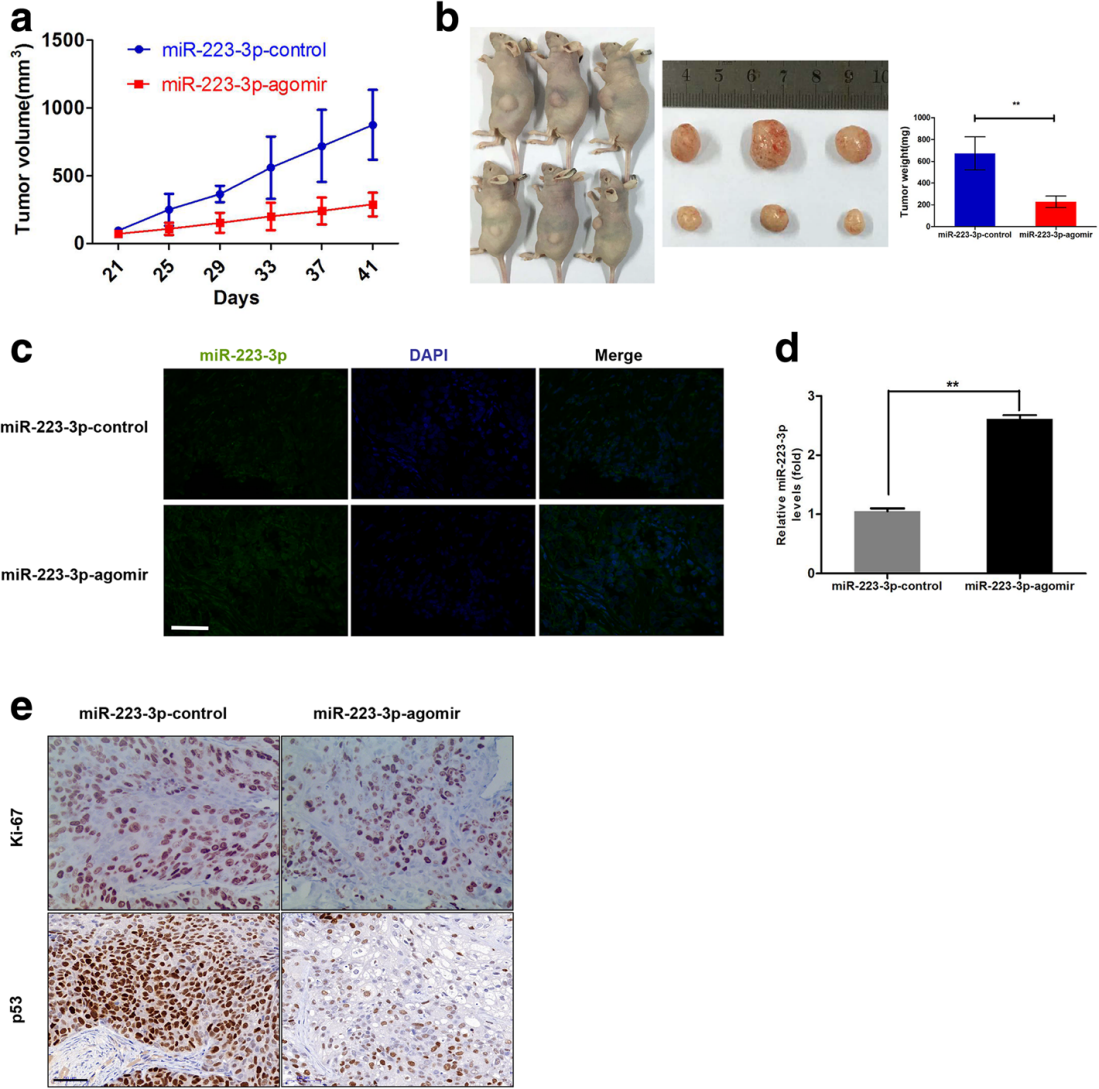
MiR-223-3p suppressed tumor growth in vivo. **a** Tumor growth curves measured after intratumoral injections with miR-223-3p agomir or control twice a week for 3 weeks. Points, mean (n = 3); bars, SD. **b** Tumor weight was significantly decreased in the miR-223-3p agomir treatment group compared with the control group. **c**, **d** qRT-PCR and ISH staining results showing that miR-223-3p was significantly upregulated in the miR-223-3p agomir treatment group compared with the control group. **e** Immunohistochemical analysis of Ki-67 and p53 in xenografts tumors of miR-NC and miR-223-3p treated groups. The scale bar was 50 μm. **P < 0.01

To explore whether the expression level of miR-223-3p affects tumorigenesis, immunodeficient female BALB/c mice were subcutaneously injected with LSCC patient–derived tumor tissues. When the tumor volume reached 50–100 mm^3^, the mice were treated with an intratumoral injection of miR-223-3p agomir or agomir control twice a week for 3 weeks. During the whole-tumor growth period, tumors from miR-223-3p agomir treatment group grew slower in comparison with the control group (Fig. 5a). After three weeks treatment, the average weight of tumors from miR-223-3p agomir treatment group was significantly smaller than that of control mice (Fig. 5b). Next, in situ hybridization (ISH) staining and qRT-PCR analysis of miR-223-3p expression were performed in resected tumor tissues. As shown in Fig. 5c and d, the expression level of miR-223-3p in miR-223-3p agomir treatment group was significantly higher than that in control group. Furthermore, immunohistochemical staining of Ki-67 to assess tumor cell proliferation revealed a reverse correlation between the miR-223-3p levels and the expression of p53 protein and cell proliferation (Fig. 5e). Such in vivo results were verified again by intratumoral injection of miR-223-3p agomir into another LSCC patient-derived tumor xenograft model (Additional file 2: Figure S2). Together, these results indicated that miR-223-3p may exert a significant inhibitory effect on tumorigenesis by repressing mutant p53 in vivo.

To sum up, our results showed that miR-223-3p expression was down-regulated in the LSCC tissues compared to that in the adjacent normal lung tissues, especially in LSCC tissues with the ability to form xenografts. Our findings demonstrated a tumor suppressive role of miR-223-3p in LSCC bearing mutant p53 in vitro and vivo. MiR-223-3p could be regulated by mutant p53 at the transcriptional level. Meanwhile, p53 is a direct target gene of miR-223-3p, suggesting miR-223-3p and mutant p53 were reciprocally linked in a feedback loop affect LSCC proliferation and metastasis.

## Discussion

Accumulating studies have described that miRNA can function as tumor suppressors or oncogenes by regulating target gene in various human cancers. Multiple miRNAs have been demonstrated to be involved in several biological processes in NSCLC, such as cell proliferation, apoptosis, invasion and metastasis. LSCC is the second most prevalent type of NSCLC. Although the microRNA profiling of NSCLC has contributed to the understanding of the biology of these cancers, the microRNA signatures identified in NSCLC (including adenocarcinoma and SCC) were not consistent among different clinical studies. Moreover, the most aberrantly expressed miRNAs were identified between tumor and normal samples [24]. As patient tumors that successfully form xenografts represented more aggressive, therefore, aberrantly expressed miRNAs between successful engraft tumors and failed engraft tumors may possess greatest potential to affect tumor proliferation and metastasis.

This study investigated and compared miRNA expression profiles between LSCC tissues that were successfully engrafted into immunocompromised mice and those that were not engrafted. Based on the evidence reported to date, it is clear that miR-223 has an impact on different cellular processes, ranging from cell cycle regulation and invasiveness to hematopoietic differentiation and immune cell function [25]. Particularly, miR-223 participates in the different processes of multiple lung diseases, such as tuberculosis [26], chronic obstructive pulmonary disease [27], lung inflammation [28], and similar. Nonetheless, miR-223 has shown to function as a tumor suppressor in Lewis lung carcinoma cells [10]. However, it may also function as an oncogene in lung adenocarcinoma A549 cells [11]. Therefore, this study focused on determining whether miR-223-3p functioned as a tumor suppressor in LSCC through in vitro and in vivo experiments; it was down-regulated in successfully engrafted tumors (Fig. 1a). MiR-223-3p was significantly downregulated in 12 LSCC tissues compared with adjacent nontumoral lung tissues and was more obvious in the XG group (Fig. 1b, c and d). Moreover, exogenous miR-223-3p could significantly affect the growth and migration of LSCC cells in vitro (Fig. 2 and Additional file 1: Figure S1). At the same time, the present study also found that miR-223-3p expression repressed tumorigenesis in vivo (Fig. 5 and Additional file 2: Figure S2). These results suggested that the downregulation of miR-223-3p might be associated with cellular mechanisms related to LSCC development.

Mutant p53 proteins are expressed at a high frequency in human tumors and are associated with cell proliferation and migration [29]. The p53 gene was sequenced and analyzed in 12 LSCC tumor tissues in this study. Among them, 6 of 12 patients harbored p53 mutations. Moreover, 5 of 6 patients harboring p53 mutations were successfully formed xenografts. Furthermore, 4 of 5 patients who successfully formed xenografts harbored p53^215C > G^ missense mutation. The results suggested xenograft engraftment was associated with p53 mutations, especially p53^215C > G^ missense mutation. Previous studies have shown that mutant p53 protein downregulates miR-223 expression in breast and colon cancer cell lines by binding miR-223 promoter and by reducing its transcriptional activity [23]. Our results also confirmed that mutant p53 directly binds to the miR-223 promoter by ChIP assay (Fig. 3a, and b) in LSCC. Down-regulation of mutant p53 in the SK-MES-1 and NCI-H520, miR-223-3p expression was up-regulated (Fig. 3c). In addition, the relative expression level of miR-223-3p significantly decreased in the LSCC tissue samples with mutant p53 (Fig. 3d). Moreover, miR-223-3p was significantly down-regulated upon mutant p53^215C > G^ upregulation in the SK-MES-1 (Fig. 3e). These observations indicated that miR-223-3p was also regulated by mutant p53 at the transcriptional level in LSCC.

Interestingly, miRNA target–predicted database showed that p53 contained 3’-UTR elements that were partly complementary to miR-223-3p. Luciferase reporter, Western blot and IHC assay confirmed that the p53 gene was regulated by miR-223-3p in LSCC (Fig. 4a, and b, Fig. 5e). In addition, we inhibited mutant p53 expression by RNA interference and found that the inhibition of p53 could significantly inhibit proliferation and migration in LSCC cells (Fig. 4c and d). Thus, it was concluded that downregulation of mutant p53 might be a mechanism by which miR-223-3p exerts its tumor suppressor functions. All together, these data indicated the existence of a regulatory network between miR-223-3p and mutant p53 proteins in LSCC cells to affect tumor proliferation and metastasis. Although we have shed a new light on the molecular mechanism responsible for miR-223-3p in LSCC progression, the other targets of miR-223-3p require further investigation. On the other hand, the other detailed mechanisms by which miR-223-3p is downregulated, such as through DNA promoter methylation [30], interaction with long noncoding RNA [31] or inflammation cytokines (such as IL-1β, TNF-α) induction [32], still need to be elucidated in future studies.

## Conclusions

Our study reported the altered miRNA expression pattern between LSCC tissues that were successfully engrafted into immunocompromised mice (XG) and those that did not engraft (no-XG) showing that miR-223-3p was significantly down-regulated in the XG group. Our findings demonstrated a tumor suppressor role of miR-223-3p in LSCC bearing p53 mutations in vitro and vivo. In addition, we demonstrated that a regulatory network existed between miR-223-3p and mutant p53 proteins in LSCC cells; mutant p53/miR-223-3p feedback loop regulated the proliferation and migration of LSCC cell lines. The present experimental data suggested that miR-223-3p plus mutant p53 may serve as an improved prognostic biomarker useful for prediction of prognosis in patients with LSCC. These data may also provide a novel strategy for treatment of patients with LSCC bearing p53 mutations.

## Additional files

Additional file 1: **Figure S1.** Downregulation of miR-223-3p promoted cell proliferation and migration in vitro. (a) Effect of miR-223-3p-inhibitor transfection into LSCC cells was confirmed using qRT-PCR. Tumor cells were transfected with miR-223-3p inhibitor or inhibitor-NC and then subjected to cell viability assay (b), colony-formation assay (c) and migration assays (d). Data are presented as the mean ± SD of three replicates. **P < 0.01; *P < 0.05. (TIF 19219 kb)

Additional file 2: **Figure S2.** MiR-223-3p suppressed tumor growth in vivo. (a) Tumor growth curves measured after intratumoral injections with miR-223-3p agomir or control twice a week for 3 weeks. (b) Tumor weight was significantly decreased in the miR-223-3p agomir treatment group compared with the control group. (c-d) qRT-PCR and ISH staining results showing that miR-223-3p was significantly up-regulated in the miR-223-3p agomir treatment group compared with the control group. The scale bar was 50 μm. (e) Immunohistochemical analysis of Ki-67 and p53 in xenografts tumors of miR-NC and miR-223-3p treated groups. The scale bar was 50 μm. **P < 0.01. (TIF 30590 kb)

## Abbreviations

LSCC: Lung squamous cell carcinoma
no-XG: Primary tumor tissues that failed to form xenografts in nude mice
NSCLC: Non-small cell lung cancer
PDTX: Patient-derived tumor xenografts
UTR: Untranslated region
XG: Primary tumor tissues that successfully formed xenografts in nude mice
