# Supplementary figures and images for: MiR-223-3p functions as a tumor suppressor in lung squamous cell carcinoma by miR-223-3p-mutant p53 regulatory feedback loop
Source: J Exp Clin Cancer Res. 2019 Feb 12;38:74. doi: 10.1186/s13046-019-1079-1 (PMC6373043; doi:10.1186/s13046-019-1079-1)

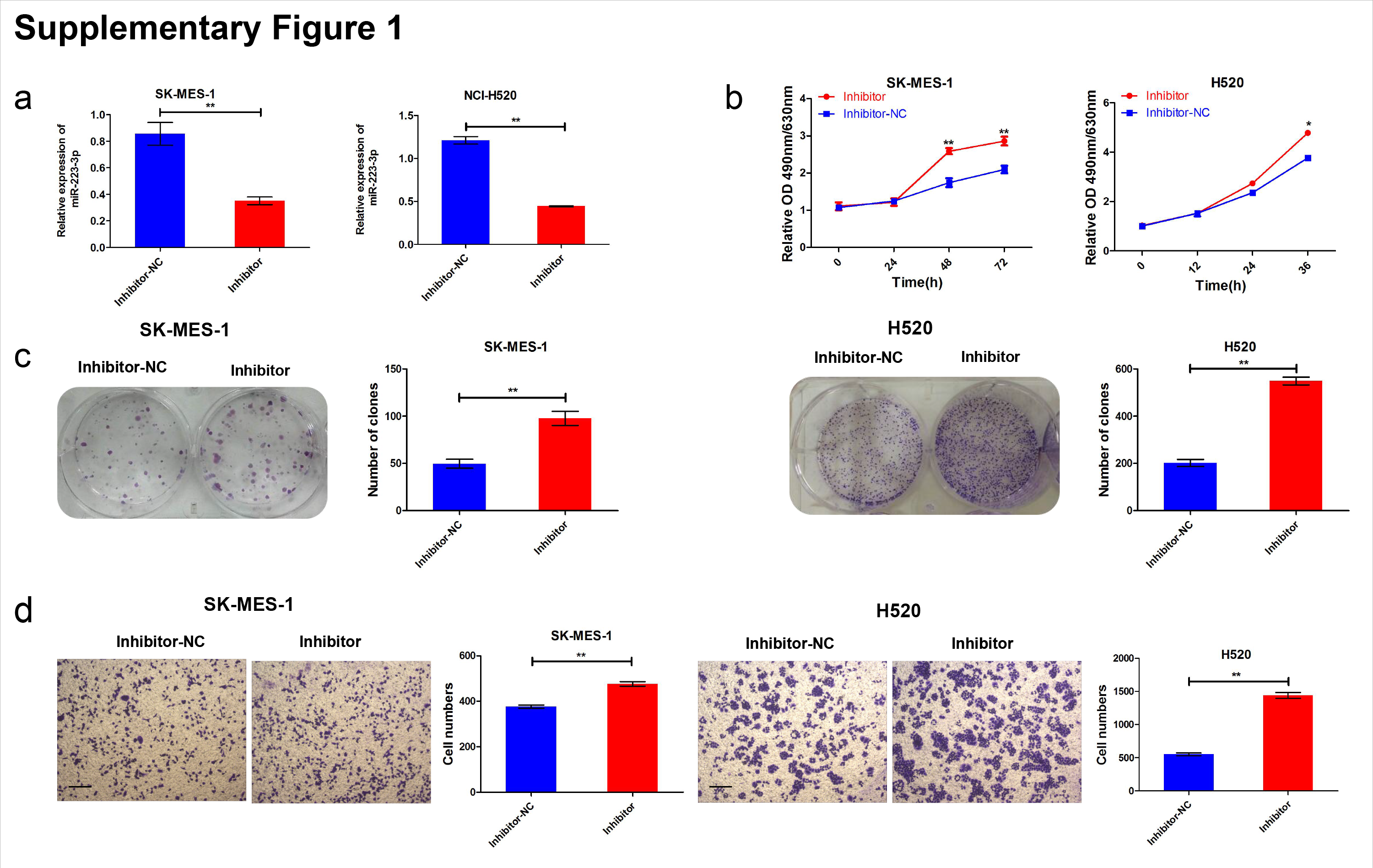

Supplement: Supplementary file 1 — Figure S1. Downregulation of miR-223-3p promoted cell proliferation and migration in vitro. (a) Effect of miR-223-3p-inhibitor transfection into LSCC cells was confirmed using qRT-PCR. Tumor cells were transfected with miR-223-3p inhibitor or inhibitor-NC and then subjected to cell viability assay (b), colony-formation assay (c) and migration assays (d). Data are presented as the mean ± SD of three replicates. **P < 0.01; *P < 0.05. (TIF 19219 kb) [file 13046_2019_1079_MOESM1_ESM.tif]

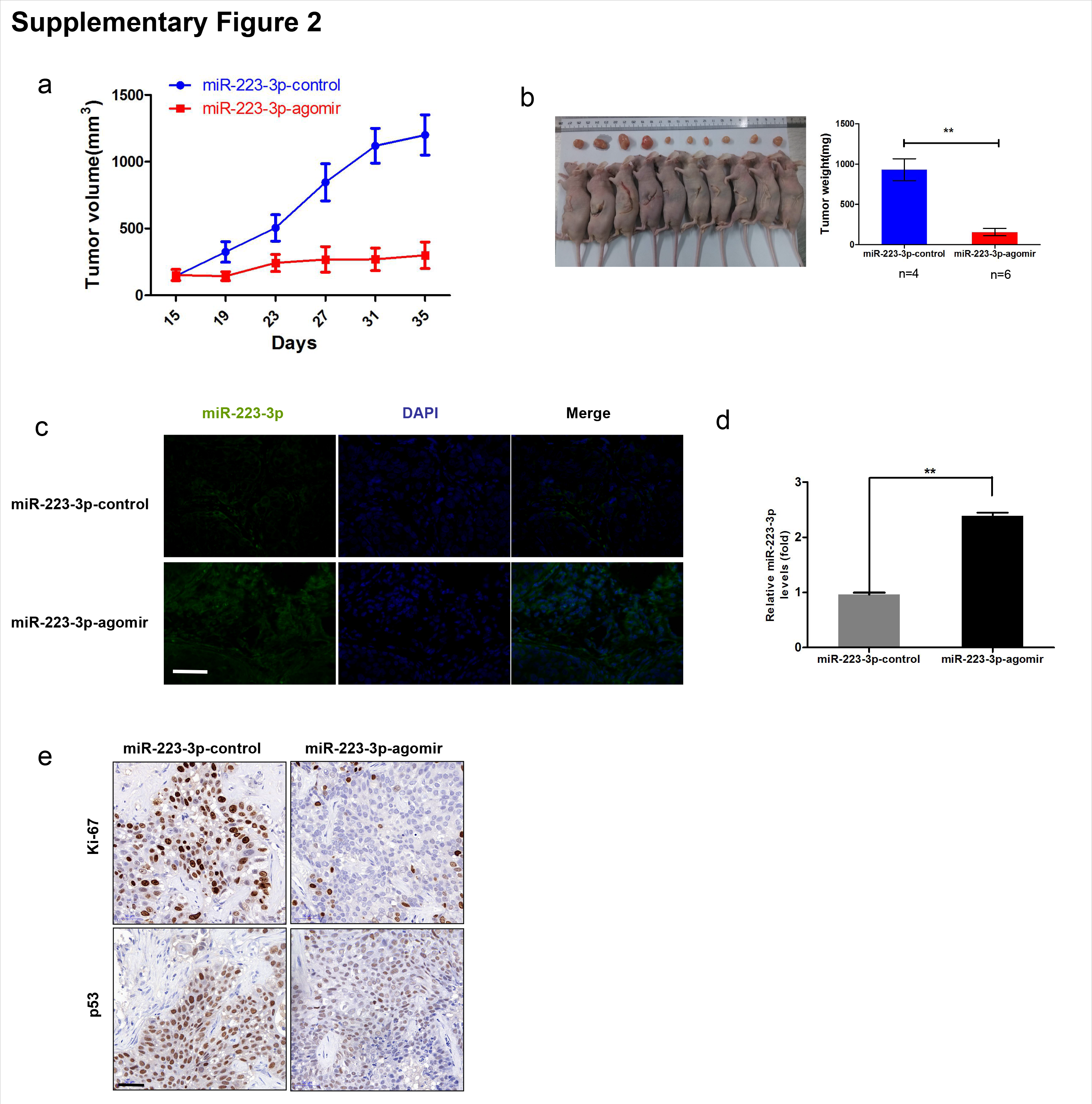

Supplement: Supplementary file 2 — Figure S2. MiR-223-3p suppressed tumor growth in vivo. (a) Tumor growth curves measured after intratumoral injections with miR-223-3p agomir or control twice a week for 3 weeks. (b) Tumor weight was significantly decreased in the miR-223-3p agomir treatment group compared with the control group. (c-d) qRT-PCR and ISH staining results showing that miR-223-3p was significantly up-regulated in the miR-223-3p agomir treatment group compared with the control group. The scale bar was 50 μm. (e) Immunohistochemical analysis of Ki-67 and p53 in xenografts tumors of miR-NC and miR-223-3p treated groups. The scale bar was 50 μm. **P < 0.01. (TIF 30590 kb) [file 13046_2019_1079_MOESM2_ESM.tif]
